# Effect of attentional load on audiovisual speech perception: evidence from ERPs

**DOI:** 10.3389/fpsyg.2014.00727

**Published:** 2014-07-15

**Authors:** Agnès Alsius, Riikka Möttönen, Mikko E. Sams, Salvador Soto-Faraco, Kaisa Tiippana

**Affiliations:** ^1^Psychology Department, Queen's UniversityKingston, ON, Canada; ^2^Department of Experimental Psychology, University of OxfordOxford, UK; ^3^Brain and Mind Laboratory, School of Science, Aalto UniversityEspoo, Finland; ^4^Institut Català de Recerca i Estudis AvançatsBarcelona, Spain; ^5^Brain and Cognition Center, Universitat Pompeu FabraBarcelona, Spain; ^6^Institute of Behavioural Sciences, University of HelsinkiHelsinki, Finland

**Keywords:** audiovisual speech perception, multisensory integration, McGurk effect, attention, event-related potentials

## Abstract

Seeing articulatory movements influences perception of auditory speech. This is often reflected in a shortened latency of auditory event-related potentials (ERPs) generated in the auditory cortex. The present study addressed whether this early neural correlate of audiovisual interaction is modulated by attention. We recorded ERPs in 15 subjects while they were presented with auditory, visual, and audiovisual spoken syllables. Audiovisual stimuli consisted of incongruent auditory and visual components known to elicit a McGurk effect, i.e., a visually driven alteration in the auditory speech percept. In a Dual task condition, participants were asked to identify spoken syllables whilst monitoring a rapid visual stream of pictures for targets, i.e., they had to divide their attention. In a Single task condition, participants identified the syllables without any other tasks, i.e., they were asked to ignore the pictures and focus their attention fully on the spoken syllables. The McGurk effect was weaker in the Dual task than in the Single task condition, indicating an effect of attentional load on audiovisual speech perception. Early auditory ERP components, N1 and P2, peaked earlier to audiovisual stimuli than to auditory stimuli when attention was fully focused on syllables, indicating neurophysiological audiovisual interaction. This latency decrement was reduced when attention was loaded, suggesting that attention influences early neural processing of audiovisual speech. We conclude that reduced attention weakens the interaction between vision and audition in speech.

## Introduction

Many events in our everyday life stimulate different sensory systems in a correlated fashion. The integration of such diversity of sensory information allows the brain to construct efficient and adaptive representations of the external world (e.g., Stein and Meredith, 1993), but the neural mechanisms underlying multisensory binding are still not well understood (e.g., van Atteveldt et al., 2014). A question under current debate is to which extent multisensory integration occurs pre-attentively or can be influenced by higher-order cognitive processes (e.g., Talsma et al., 2010).

Speech perception is one of the classical examples of multisensory binding in humans, whereby acoustic information is combined with the sight of corresponding facial articulatory gestures. Audiovisual association of facial gestures and vocal sounds has been demonstrated in non-human primates (Ghazanfar and Logothetis, 2003) and in pre-linguistic children (e.g., Kuhl and Meltzoff, 1982; Burnham and Dodd, 2004; Pons et al., 2009), arguing for the existence of an early basis of this capacity (Soto-Faraco et al., 2012). One striking demonstration of multisensory binding in speech is the McGurk effect (McGurk and MacDonald, 1976), which results from exposure to mismatched acoustic and visual signals, often leading observers to hear an illusory speech sound. For example, when the sound of [ba] is dubbed onto a video clip containing the articulatory movements corresponding to [ga], the observer usually experiences hearing a fusion between the acoustic and the visual syllable, e.g., [da] or [tha], or even the visually specified [ga]. Discrepant visual speech thus alters the auditory speech percept, and may even dominate it, e.g., a visual [da] dubbed onto an acoustic [ba] is often heard as [da], and a visual [na] dubbed onto an acoustic [ma] is heard as [na] (MacDonald and McGurk, 1978; for a detailed discussion on the definition of the McGurk effect, see Tiippana, 2014). The compelling phenomenology of the McGurk illusion has been often used as an argument supporting the effortless and mandatory (i.e., unavoidable) nature of multisensory integration in speech (e.g., Rosenblum and Saldaña, 1996; Soto-Faraco et al., 2004).

Several recent studies have, however, put into question the impenetrability of audiovisual integration to attentional modulation, both in the speech (Tiippana et al., 2004, 2011; Alsius et al., 2005, 2007; Soto-Faraco and Alsius, 2007, 2009; Andersen et al., 2009; Fairhall and Macaluso, 2009; Alsius and Soto-Faraco, 2011; Buchan and Munhall, 2011, 2012) and the non-speech domains (e.g., Senkowski et al., 2005; Talsma and Woldorff, 2005; Fujisaki et al., 2006; Talsma et al., 2007). Of particular interest for the current study, Alsius et al. (2005) tested to which extent audiovisual speech perception could be modulated by attentional load. They varied the amount of available processing resources by measuring the participants' susceptibility to the McGurk effect in a Single vs. Dual task paradigm. In the Dual task condition, participants were instructed to perform a very demanding detection task on rapidly presented visual or auditory streams, while repeating back the words uttered by a speaker (which were dubbed to obtain the McGurk effect). In the Single task condition, participants were shown the same displays but just prompted to repeat back the words. In the Dual task condition, the percentage of illusory McGurk responses decreased dramatically. That is, when the load was high, and thus processing resources presumably depleted, participants became less susceptible to experience the McGurk effect than when they had spare processing resources.

Effects of attention on multisensory processing have been reported also outside the domain of speech, for example using event-related potentials (ERPs). Talsma and Woldorff (2005; see also Senkowski et al., 2005; Talsma et al., 2007) showed that the difference usually found between the evoked potentials to audiovisual (AV) events and the sum of unisensory events (A+V; “additive model”) was larger at attended than unattended locations of space. This modulation was seen both in short and long latency ERP components. Talsma et al.'s (2007) study suggests that spatial attention affects the early sensory integration of simple (non-speech) multisensory events. It remains unknown, however, how attentional load (as in Alsius et al., 2005) modulates the neural mechanisms underlying audiovisual speech integration.

Electrophysiological studies within the speech domain have consistently shown that visual speech can modify activity in the auditory cortex during audiovisual speech perception as early as ~100–200 ms post-stimulus (Sams et al., 1991; Colin et al., 2002; Möttönen et al., 2002, 2004; Klucharev et al., 2003; Besle et al., 2004; Van Wassenhove et al., 2005). There are a variety of electrophysiogical markers of audiovisual interactions in speech (e.g., Saint-Amour et al., 2007; Bernstein et al., 2008; Ponton et al., 2009; Arnal et al., 2011). Although these markers are not exclusive of audiovisual speech (Stekelenburg and Vroomen, 2007), they are thought to reflect important aspects of the speech perception process such as cross-modal prediction and phonological processing (Brunellière et al., 2013).

One of the best-known electrophysiological correlates of audiovisual interactions in speech is temporal facilitation of the N1/P2 component of the auditory ERPs (Van Wassenhove et al., 2005; Baart et al., 2014; Knowland et al., 2014). Some studies have also found an amplitude reduction of the N1/P2 complex in audiovisual speech contexts (Klucharev et al., 2003; Besle et al., 2004; Van Wassenhove et al., 2005; Pilling, 2009; Knowland et al., 2014), but this effect has not always been replicated (Miki et al., 2004; Möttönen et al., 2004; Baart et al., 2014). It is also relevant here to note that studies on the effect of attention on the auditory evoked potentials have often focused on modulations within the N1 and P2 time windows, generally demonstrating an amplification of these ERP components when the stimulus is under the focus of attention (see Hillyard et al., 1973; Picton et al., 1974; Näätänen, 1982 for seminal studies).

The goal of the present study was to characterize the role of attentional load in audiovisual integration of speech, capitalizing on the electrophysiological marker of temporal facilitation. The amount of processing resources directed to audiovisual stimuli was manipulated by using a Single vs. Dual task paradigm adapted from Alsius et al. (2005, 2007). ERPs were recorded while participants were presented with audiovisual spoken syllables known to produce the McGurk effect, as well as unisensory auditory and visual syllables. These were interspersed within an Rapid Serial Visual Presentation (RSVP) of line drawings. In the Single task condition, participants were asked to identify some of the syllables regardless of the RSVP, whereas in the Dual task condition, participants were asked to perform the syllable identification task and, in addition, to detect repetitions in the RSVP.

We expected that audiovisual interaction would modulate the N1/P2 component complex of the auditory ERPs in the Single task condition, as shown in previous studies (e.g., Van Wassenhove et al., 2005; Baart et al., 2014; Knowland et al., 2014). Crucially, with respect to the attentional load, we hypothesized that these modulations would be reduced or eliminated in the Dual task condition if early audiovisual interactions in the auditory cortex are influenced by attention demands. We thus predicted that the temporal facilitation of the N1/P2 complex for audiovisual ERPs would be smaller in the Dual than Single task condition.

## Methods

### Participants

Sixteen healthy right-handed participants, native speakers of Finnish, participated in the experiment. Data from two participants were excluded from the analyses because of excessive artifacts in EEG signals. In the remaining 14 participants, the mean age was 22 years (range 19–28 years; 3 female). Participants reported normal audition and normal or corrected-to-normal vision. All of them gave their informed consent to participate in the study. The study was conducted in accordance with the principles expressed in the Declaration of Helsinki, and adhered to the guidelines of the American Psychological Society and the ethical policies of Helsinki University of Technology (currently Aalto University; please note that at the time of data collection, there was no ethical committee at the university from which to apply for approval).

### Stimuli

Digital video recordings of a Finnish female speaker (black-and-white, full-face) uttering the syllables [mi] and [ni] were edited with Studio Purple software and transformed to bitmap sequences. The image contrast was lowered to minimize visual ERP responses. The auditory components of the syllables were saved as 16 Bit—44.1 kHz waveform audio format (WAV) files. The auditory unisensory trials consisted of an acoustic syllable [mi] or [ni] combined with a still image of the talker's face with the lips closed. The visual unisensory trials consisted of the silent presentation of the speaker's articulation of the [mi] or [ni] syllable (presented as a sequence of still images, 25 frames per second). The McGurk-type audiovisual trials were created by temporally aligning the acoustic burst onset of the auditory syllable [mi] to the burst onset of the visual [ni]. This particular combination is known to elicit an auditory percept dominated by the visual information so that observers usually hear /ni/ (MacDonald and McGurk, 1978; Tiippana et al., 2011, where the same stimuli were used as here). Each visual syllable was presented in a clip of 600 ms duration (15 frames), and each auditory syllable lasted 265 ms. In the audiovisual stimuli, the auditory syllable started 215 ms after the onset of visual articulatory gestures (5th frame).

There were two experimental conditions run in different blocks (Single task and Dual task condition, see Procedure). Each block contained a sequence of a total of 180 audiovisual (AV) syllables presented in random order (120 McGurk stimuli, 30 congruent [mi], 30 congruent [ni]), 150 visual-only (V) syllables (120 [ni], 30 [mi]), and 150 auditory-alone (A) syllables (120 [mi], 30 [ni]). The inter-syllable interval was chosen randomly between 1200 and 3600 ms (in order to minimize anticipatory slow waves) contained a still picture of the talkers face. After ~10% of the syllables (a total of 10 times per stimulus-type, in each condition) and distributed randomly in the sequence, the question “What did you hear?” appeared on the screen, prompting participants to make an identification response on the last syllable presented. The syllable sequence was interspersed within a RSVP stream of line drawings of common objects presented in between syllables (3–6 drawings at each inter-syllable period), and superimposed on the still image of the talker's face. The RVSP stopped while syllables were presented in order to prevent overlapping ERPs to pictures and syllables. Nevertheless, monitoring had to be sustained across these breaks because repetitions could straddle syllable presentations.

In the RSVP, each drawing was presented for 60 ms, stimulus onset asynchrony (SOA) varied randomly between 400 and 600 ms, and they roughly covered the distance between the upper part of the speaker's lips and the nose. Each drawing in the sequence was chosen at random from a set of 105 different drawings from the Snodgrass and Vanderwart (1980) picture database, and rotated in one of three possible different orientations (45, 90, or 135°, equiprobably). Picture repetitions (i.e., targets in the Dual task condition) occurred on average every seven stimuli, and could occur within or across the inter-syllable period.

The stimulus presentation protocol was controlled using Presentation software (Neurobehavioural system, Inc.). Images were presented using a 19″ CRT monitor. Sounds were delivered at an overall intensity of 65 dB(A) SPL through two loudspeakers positioned on both sides of the monitor.

### Procedure

**Figure 1:**
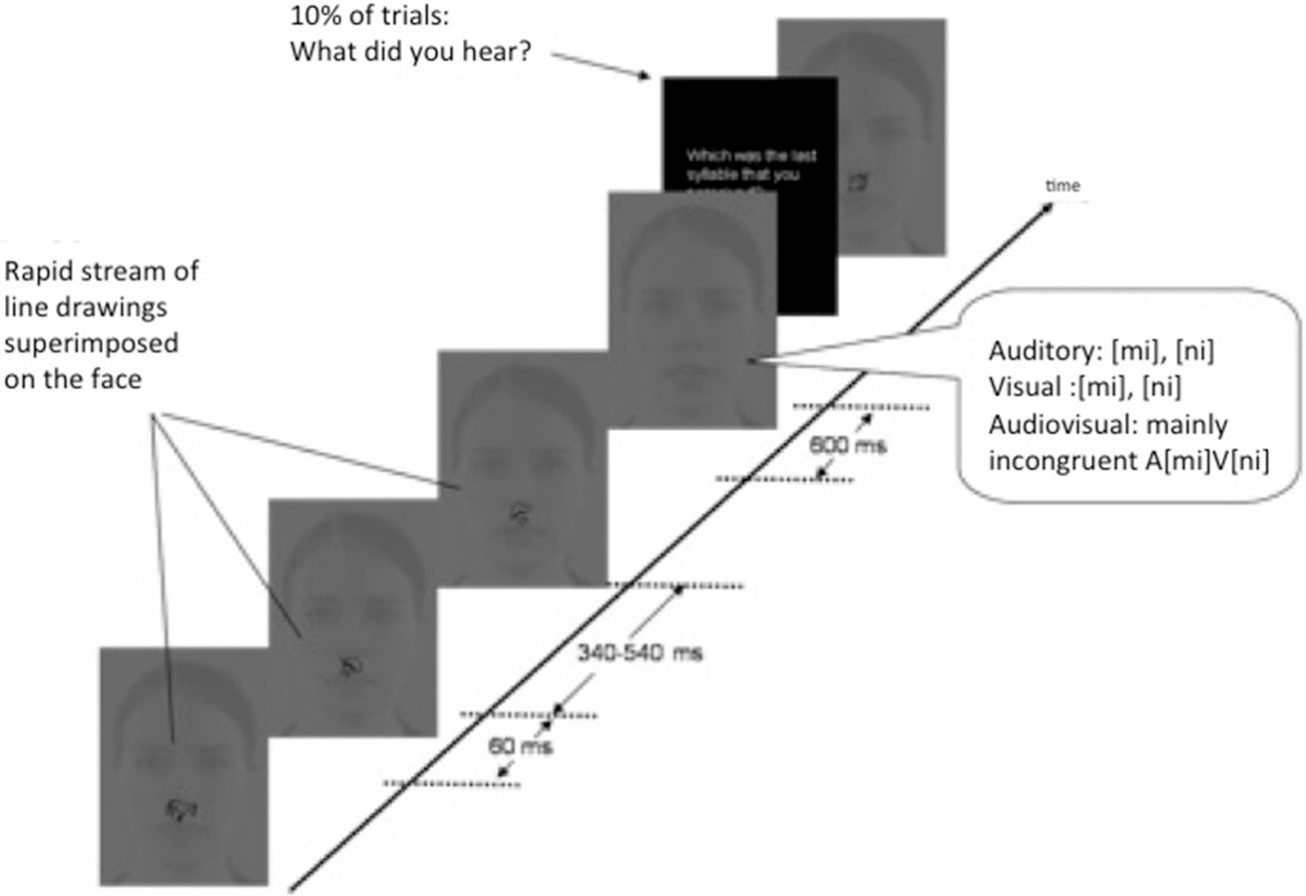
**Setup**. A Single vs. Dual task paradigm was used to divert attention from spoken syllables. In the Single task condition participants reported the syllables that the speaker said (10% of trials), whereas in the Dual task they were asked, in addition, to monitor a Rapid Visual Serial Presentation stream of line drawings for repeated items. The syllables were presented audiovisually, auditorily, and visually.

Participants sat 1.1 m from the monitor on a comfortable armchair placed in an electrically and acoustically shielded room. They were instructed to make a syllable identification response when prompted to (on ~10% of the trials) by pressing the corresponding key on the keyboard (labeled “mi” or “ni”). The amount of available processing resources directed to the spoken syllables was manipulated by the instructions regarding a concurrent task. Whereas in the Single task condition participants just had to identify the syllable when prompted, in the Dual task condition participants were asked to, in addition to the identification response, continuously monitor the RSVP of line drawings superimposed on the image of the talker's face for repetitions, and respond by pressing a key labeled “X” on the keyboard when repetitions occurred (see Figure 1). All participants were tested in both the Dual and the Single task condition. The order of the tasks was counterbalanced between participants. A training block was performed before starting each task.

### EEG data acquisition

EEG recordings were made using BrainVision software with 20 silver/silver chloride electrodes (BrainCap, Brainproducts) mounted on an elastic cap (reduced 10–20 system: Fp1, Fp2, C3, C4, P3, P4, O1, O2, F7, F8, T7, T8, Fz, Cz, Pz, TP9, TP10). Two additional electro-oculogram electrodes (Eog1 and Eog2) were placed above and below eyes in order to detect blink artifacts, and one electrode was attached to the tip of the nose in order to provide a common reference channel. Prior to each session, all electrode impedances were set below 10 kΩ. EEG data were recorded with a sampling frequency of 500 Hz.

### Data analysis

ERPs were averaged offline separately for the three stimulus types (auditory [mi], visual [ni], and audiovisual McGurk stimulus A[mi]+V[ni]) using Vision Analyzer software. The tip of the nose was selected as the reference for the analysis. Data were filtered using a bandpass of 1–40 Hz (attenuation 24 dB/octave) and segmented in time windows of −100 to 400 ms relative to the auditory onset of the syllable (i.e., the zero time corresponding to the onset of the sound, or the onset of the 5th video frame for the visual-only trials). A 100-ms pre-stimulus (before the auditory onset) baseline was used. Trials with signal amplitudes exceeding 100 μV at any electrode within the −100 to +400 ms window were automatically rejected to eliminate response contamination by eye movements or muscular activities. Trials in which a motor response was produced to any of the two tasks at any time between 100 ms prior to 400 ms after the syllable was presented were also excluded from the ERP analyses. The averaged ERPs for each subject and condition contained a minimum of 100 epochs after trial rejection. In order to ensure sufficient number of observations, the EEG session was extended when the number of artifacts detected during the experiment was high.

#### Estimation of AV interactions

AV interactions were assessed by using a modified version of a commonly used additive model: AV-[A+V] (Stein and Meredith, 1993; Giard and Peronnet, 1999; Molholm et al., 2002; Teder-Sälejärvi et al., 2002; Klucharev et al., 2003; Besle et al., 2004; Möttönen et al., 2004). As we specifically focused on the modulation of auditory ERPs, which have been shown to be prominent during audiovisual speech processing, we compared the ERPs evoked by the unisensory auditory stimulus (A) with the subtraction between the ERPs evoked by the audiovisual (AV) and visual (V) stimuli, i.e., AV-V (Baart et al., 2014). The AV-V wave represents the EEG activity evoked by the audiovisual syllables without the contribution of the visual component. Differences between the AV-V wave and the A wave should reveal how audiovisual interaction affects N1 and P2 in Single and Dual task conditions.

The A and AV-V waveforms were statistically compared by performing sample-by-sample (~2 ms steps) sequential paired Student *t*-tests and by comparing the peak latencies and amplitudes of the N1 and P2 components of the auditory ERPs in both Single and Dual task conditions. The sample-by-sample student *t*-tests were performed from audio onset to 300 ms post-audio onset in all electrodes for the data from the Single and the Dual task conditions. In order to reduce the likelihood of false-positives due to a large number of *t*-tests, we considered differences to be significant when the *p* values were lower than 0.05 at 10 (= 20 ms) or more consecutive time points (Guthrie and Buchwald, 1991; see also Molholm et al., 2002; Besle et al., 2004 for the same analysis procedure).

The Fz electrode was selected for comparison of A and AV-V. Electrode selection was necessary since in many electrodes the RSVP elicited more pronounced and longer-lasting ERPs during the Dual than Single task condition, which could contaminate the baselines to the speech stimuli. In the Fz recording site, the baseline was not contaminated, the N1 and P2 responses to A stimuli were the strongest, and the differences between A and AV-V were maximal.

The N1 peak was defined as the largest negative peak occurring between 65 and 165 ms after the auditory onset at Fz from A and AV-V ERPs. The P2 peak was computed as the highest positive value in a temporal window of 135–285 ms after the onset of the auditory stimulus. After semi-automatic detection of the peaks, two experimenters blind to the subject's condition visually revised that each detected peak had been correctly identified.

## Results

### Behavioral results

#### Syllable identification

**Figure 2:**
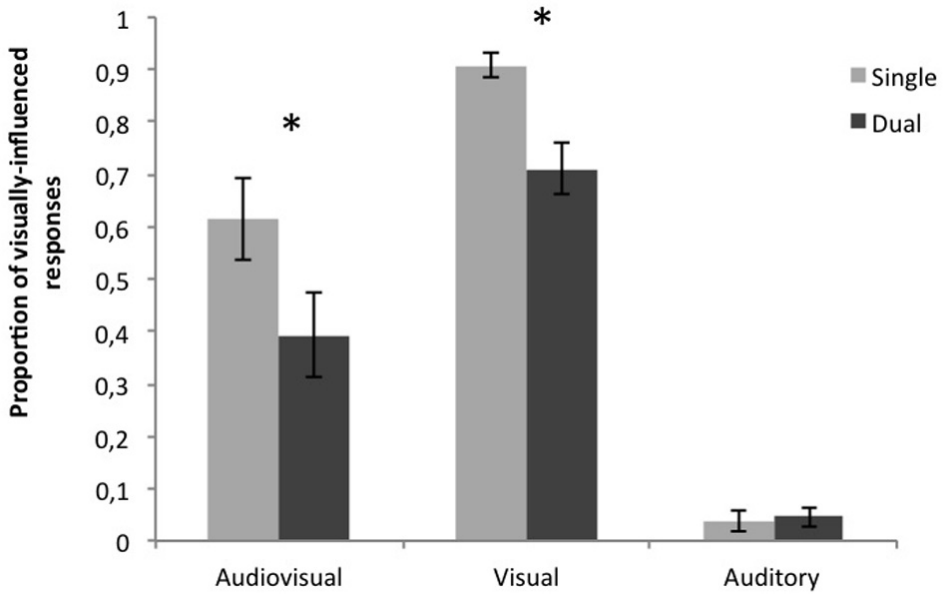
**Proportions of visually-influenced responses in the behavioral task for audiovisual, visual and auditory stimuli in Single and Dual task conditions**. For audiovisual, and visual stimuli, the proportion of visually-influenced responses (i.e., correct responses to V stimuli and “ni” responses to AV stimuli) was significantly reduced in the Dual task condition. The asterisk denotes significant differences (*p* < 0.05).

For each stimulus type (AV, V, A) we assessed the proportion of visually-influenced responses. The data were submitted to repeated measures ANOVA with two within-participants factors: Stimulus type (AV, V, A) and Task (Single, Dual). The main effects of Task and Stimulus type were both significant [*F*_(1, 13)_ = 23.49, *p* < 0.001; *F*_(2, 26)_ = 20.11, *p* < 0.001, respectively] and so was the interaction between them [*F*_(2, 26)_ = 8.85, *p* = 0.001]. When each stimulus type was analyzed separately, significant effect of the Task was observed for both AV and V stimuli (*t* = 4.1, *p* = 0.001 and *t* = 4.4, *p* = 0.001, respectively), but it did not affect the identification of A stimuli (*t* = 0.00, *p* = 1). That is, the percentage of participants' visually-influenced responses was significantly lower in the Dual than Single task condition for audiovisual and visual stimuli. No difference was found in the size of this decrease between AV and V [*F*_(1, 13)_ = 0.18, *p* = 0.68; Figure 2]. These results mean that the McGurk effect was weaker and speechreading poorer in the Dual than Single task condition.

#### Target detection in the concurrent task of the Dual task condition

In the concurrent repetition task (Dual task condition), the overall hit rate (detection response within 2 s after a target occurred in the RSVP stream) was 0.35 (note that the average probability of target occurrence was 1 every 7), and false alarm rate (erroneously responding when no target occurred within the previous 2 s) was 0.008.

### Electrophysiological results

#### Audiovisual interactions in the single task condition

**Figure 3:**
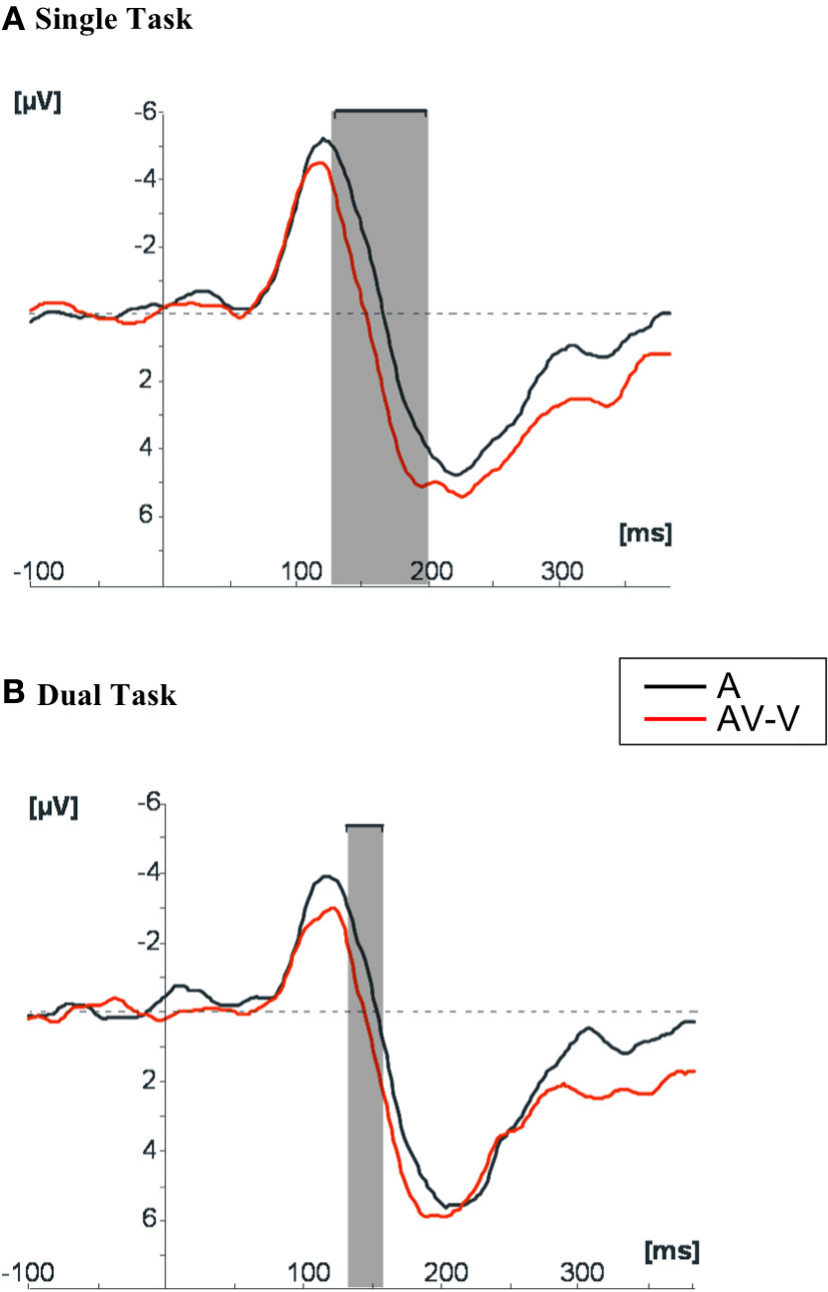
**Grand average of the ERPs to auditory stimuli in comparison with the ERPs resulting from the subtraction AV-V at Fz, in (A) Single task condition and (B) Dual task condition**. The shaded areas indicate the time windows in which the two ERPs differed significantly (*p* < 0.05) in amplitude.

Figure 3A shows the grand-average ERPs to the A stimuli and the AV-V difference wave at Fz in the Single task condition. In the early time window (100–140 ms) both responses were characterized by the typical negative N1 component originating in the auditory cortex (Vaughan and Ritter, 1970; Picton et al., 1974). N1 was followed by a P2 component. Paired sequential *t*-tests showed a reliable difference between AV-V and A ERPs from 130 to 200 ms (all *p* < 0.05) after the auditory onset. This was because of the earlier occurrence of the N1 offset and P2 onset in the AV-V wave than in A, suggesting that auditory responses were speeded up by the presentation of concurrent visual speech information (see peak latency analysis below).

**Figure 4:**
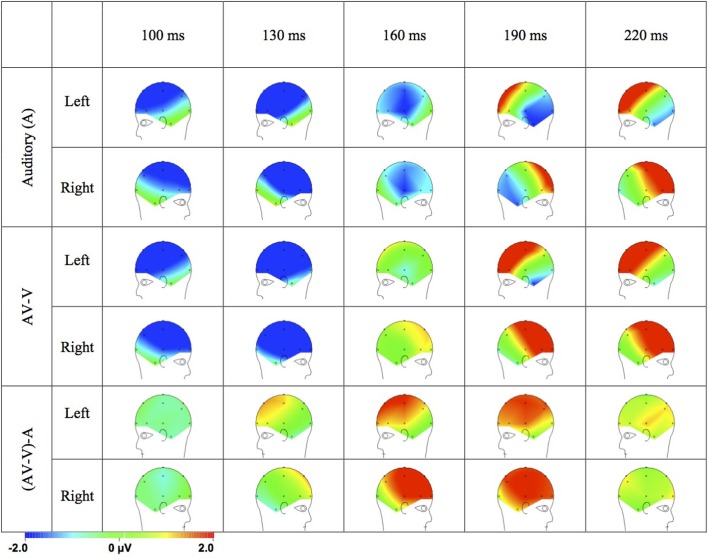
**Topographical distributions of the grand average ERPs for the auditory stimuli and AV-V and the (AV-V)-A difference waves in time steps of 30 ms**.

The topographical distribution of the ERPs to the A, AV-V, and (AV-V)-A difference wave (Figure 4) support the assumption that the difference between A and AV-V ERPs was due to modulation of auditory ERPs. In the ERPs to A stimuli, N1 peaked at 122 ms and was maximal at fronto-central sites (Fz: −5.670 μ V) with a polarity inversion at the mastoids (TP9: 0.659 μ V; TP10: 0.649 μ V). The auditory P2 peaked at 221 ms at Fz (5.76 μ V) with a polarity inversion at the mastoids (TP9: −0.79 μ V; TP10: −0.30 μ V). These distributions of ERPs to acoustic stimuli can be attributed to dipolar current sources in the auditory cortex (Vaughan and Ritter, 1970; Scherg and Von Cramon, 1986). The distributions of AV-V ERPs resembled those of the ERPs to unisensory A stimuli, suggesting similar neural generators. That is, N1 peaked at 114 ms and was maximal at Fz (−4.99 μ V) with the minimal negativity observed at mastoids (TP9: −0.377 μ V; TP10: −0.512 μ V) and P2 peaked at 204 ms at Fz (6.12 μ V) and showed reversed polarity at mastoids (TP9: −1.25 μ V; TP10: −0.17 μ V).

Importantly, the scalp distribution of the (AV-V)-A difference (see time points 160 and 190 ms in Figure 4) was similar to that of the P2 response to A stimuli (see time points 190 and 220 ms in Figure 4). The difference (AV-V)-A was also maximal at fronto-central scalp sites with polarity inversion at the mastoids (see time points 160 and 190 in Figure 4). Thus, the cerebral sources of the interaction term (AV-V)-A are likely to be similar to the ones of the auditory ERPs, suggesting that the neural generators of auditory ERPs in the auditory cortices were modulated by audiovisual interaction.

#### Effect of processing load on audiovisual interactions (Single vs. Dual task conditions)

Figure 3B shows the grand average ERPs at Fz obtained to the presentation of auditory stimuli and the AV-V difference wave in the Dual task condition. The difference between A and AV-V in the Dual task condition was significant during a short 20-ms time window (135–155 ms), compared to the 70-ms time window (130–200 ms) in the Single task condition. The difference between A and AV-V in Single and Dual tasks could not be attributed to amplitude differences, since repeated measures ANOVAs for the peak amplitudes of N1 and P2 with Modality (A, AV-V) and Task (Single, Dual) as factors showed no significant main effects or interactions.

In order to further test whether visual speech speeded up auditory processing in Single and Dual task conditions, we performed separate repeated measures ANOVAs for the peak latencies of N1 and P2 with Modality (A, AV-V) and Task (Single, Dual) as factors. Because we wanted to test a directional hypothesis that temporal facilitation should be smaller in the Dual than Single task condition, we also carried out planned comparisons (*t*-tests) on the contrast A > (AV-V) in Dual and Single task conditions for both N1 and P2.

**Figure 5:**
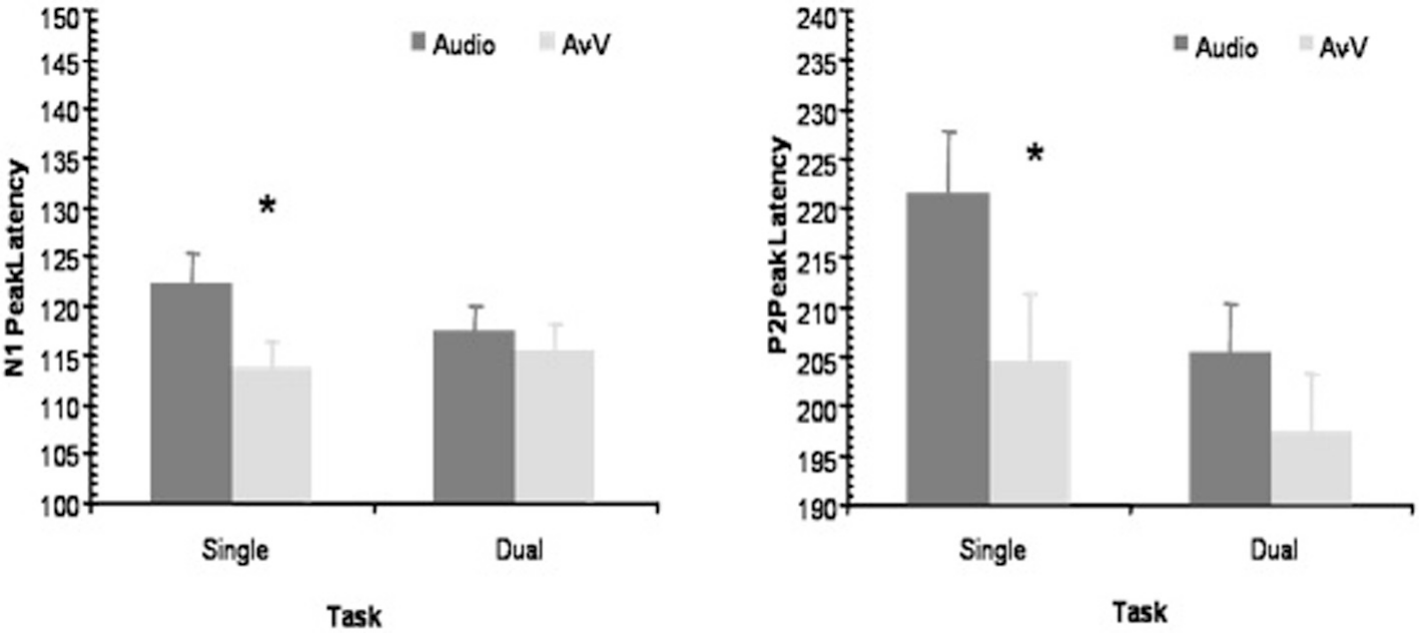
**Peak latency modulations**. The peak latencies of N1 and P2 at Fz were significantly reduced in the AV-V signal in comparison to those evoked by auditory stimuli in the Single task condition, but not in the Dual task condition. The asterisk denotes significant differences between A and AV-V (*p* < 0.05).

The main effect of Modality was significant for both N1 [*F*_(1, 13)_ = 5.92, *p* < 0.05] and P2 [*F*_(1, 13)_ = 7.01, *p* < 0.05], but the main effect of Task was not [N1: *F*_(1, 13)_ = 0.229, *p* = 0.64; P2: *F*_(1, 13)_ = 3.67, *p* = 0.08], nor was the interaction [N1: *F*_(1, 13)_ = 1.96, *p* = 0.184; P2: *F*_(1, 13)_ = 1.25, *p* = 0.0284]. The main effect of Modality arose because the latencies were overall shorter in AV-V than A for both task conditions (Dual, Single) and ERP components (N1, P2) (Figure 5).

The planned comparisons, testing the hypothesis that temporal facilitation decreased when processing resources are loaded, showed that the latency shifts between A and AV-V modalities were statistically significant only in the Single Task condition. That is, N1 peaked earlier in AV-V than in A [114 and 122 ms, respectively; *t*_(13)_ = 2.34, *p* < 0.05] in the Single Task condition, whereas in the Dual task condition the latency shift of N1 was not significant [115 and 117 ms, respectively; *t*_(13)_ = 0.804, *p* = 0.436]. In a similar fashion, P2 peaked significantly earlier in AV-V than in A [204 and 221 ms, respectively; *t*_(13)_ = 2.34, *p* < 0.05] in the Single task condition, but P2 latency shift was not significant in the Dual task condition [197 and 205 ms, respectively; *t*_(13)_ = 1.67, *p* = 0.118]. That is, when participants focused attention on a difficult unrelated visual task, the temporal facilitatory effects on the N1/P2 complex tended to be reduced or to disappear. Probably, the fact that in all cases, the AV-V latency peaks were numerically shorter than the A peaks prevented the interaction term of the ANOVA to reach significance between Task and Modality, a tendency that was nevertheless captured by the individual *t*-tests. Thus, these results are well in line with the predicted effect of attention on AV speech processing, but the conclusions (based on the *t*-tests) must be qualified by the fact that the overall ANOVAs did not reveal significant interactions.

## Discussion

To evaluate the role of attention in audiovisual speech perception, we measured behavioral and electrophysiological responses to audiovisual, auditory and visual speech stimuli in a Single vs. Dual task paradigm. Results from both measures converged to the idea that increasing demands on visual attentional resources exerted a detrimental effect on the outcome of multisensory speech processing.

The behavioral results showed that the McGurk effect was weaker in the Dual than Single task condition, showing an attentional effect on audiovisual speech perception, in agreement with previous results (Tiippana et al., 2004, 2011; Alsius et al., 2005, 2007; Soto-Faraco and Alsius, 2007, 2009; Andersen et al., 2009; Alsius and Soto-Faraco, 2011; Buchan and Munhall, 2011, 2012). However, note that at variance with the results of Alsius et al. (2005; see also Alsius et al., 2007), the identification of visual stimuli was poorer in the Dual than Single task condition. Thus, the attention effect in this study could in principle be attributed to a modulation exerted by visual attention on a modality-specific stage, interfering with the processing of visual speech prior to multisensory integration (Massaro, 1998; Tiippana et al., 2004). This interpretation has to be put under the light of electrophysiological and other recent evidence highlighting the flexible nature of the interplay between multisensory integration and attention. Indeed, there is a variety of possible stages and mechanisms enabling multisensory integration and, therefore, the impact of attention in integration processes might express in different ways (Talsma et al., 2010; van Atteveldt et al., 2014).

Our electrophysiological results replicated the previous finding (Van Wassenhove et al., 2005; Baart et al., 2014; Knowland et al., 2014) that the latency of the N1/P2 complex is reduced for audiovisual compared to auditory speech stimuli. This suggests that the visual component of audiovisual speech speeds up processing of the acoustic input, possibly in the auditory cortex (Van Wassenhove et al., 2005). When comparing peak latencies in the Single and Dual task conditions, the AV-V signal peaked significantly earlier than the A signal in the Single task condition, in which the processing resources could be fully devoted to audiovisual stimuli. Yet, when participants' processing resources were diverted to a concurrent visual task in the Dual task condition, the latency difference between the AV-V and A ERPs was non-significant. It should be noted, though, that no significant interaction between Modality and Task was found. This lack of interaction is likely to be due to the presence of some integration effect in both Single and Dual task conditions, and it advises for some caution in the interpretation of the results. Yet, what is clear is that, when tested for the specific prediction that the temporal facilitation for audiovisual ERPs would be smaller in the Dual than Single task condition, the prediction was confirmed since the facilitation was significant in the Single, but not in the Dual task condition. Supporting this conclusion, the window of significant differences between AV-V and A in the sample by sample analyses was larger in the Single Task condition (70 ms) than in the Dual Task condition (20 ms).

The electrophysiological temporal facilitation was beyond any unisensory effect since in the model used here (A vs. AV-V), any attentional effects on visual processing should have been canceled out when subtracting the visual ERPs from the audiovisual ERPs, and therefore can be ruled out as a cause of the differences. Based on the polarity and scalp topography of the difference (AV-V)-A—which was maximally positive over the fronto-central regions of the scalp and inverted in polarity in the mastoids—it is likely that the audiovisual interaction effect stems from modulation of auditory processing. This interaction, observed in the Single task condition and found to be sensitive to attentional load in the Dual task condition, was likely to be generated in the auditory cortices. The current ERP evidence thus lends some support to the view that taxing processing resources may interfere with multisensory interactions in the auditory cortex to some extent.

In absolute terms, the latency values were highest for auditory stimuli in the Single task condition. However, we think that the safest way to interpret the present pattern of results is in relative terms, not in absolute ones. This is because the baseline modulation produced by attention onto each modality separately might not be the same. Therefore, the focus should be on how AV-V peak latencies change with respect to the “default” A latency, within each attention condition. This comparison revealed a decrease in the Single, but not in the Dual task condition.

From a functional perspective, our results are in keeping with the notion that during speech perception, the auditory and visual sensory systems interact at multiple levels of processing (Schwartz et al., 1998; Nahorna et al., 2012; Barrós-Loscertales et al., 2013), and that top-down modulatory signals can influence at least some of these levels. Multisensory links do not solely rely on feed-forward convergence from unisensory regions to multisensory brain areas, but also implicate back-projections from association areas to multiple levels of (early) sensory processing that are based on current task demands (Calvert et al., 1999, 2000; Macaluso et al., 2000; Friston, 2005; Driver and Noesselt, 2008). This kind of recurrent architecture naturally allows for an integral role of attention during multisensory integration (Driver and Spence, 2000; Frith and Driver, 2000; Talsma et al., 2010; van Atteveldt et al., 2014).

Given the current evidence, briefly sketched above, we argue that since attention can influence processing at multiple levels, visual attentional load can interfere with unisensory visual processing involved in speechreading, resulting in poorer identification of visual speech, as well as with multisensory integration even at early processing stages, resulting in reduced temporal facilitation of auditory evoked potentials by audiovisual speech.

In conclusion, the present results provide new insights into the cognitive and neural mechanisms underlying audiovisual speech integration, as they suggest that visual processing load can modulate early stages of audiovisual processing. Our findings further challenge the view that audiovisual speech integration proceeds in a strictly bottom-up sensory-driven manner, independently of attention.

### Conflict of interest statement

The authors declare that the research was conducted in the absence of any commercial or financial relationships that could be construed as a potential conflict of interest.
